# Deep Reinforcement Learning-Based Adaptive Scheduling for Wireless Time-Sensitive Networking

**DOI:** 10.3390/s24165281

**Published:** 2024-08-15

**Authors:** Hanjin Kim, Young-Jin Kim, Won-Tae Kim

**Affiliations:** 1 Future Convergence Engineering Major, Department of Computer Science and Engineering, Korea University of Technology and Education, Cheonan-si 31253, Republic of Korea; gks359@koreatech.ac.kr; 2 Department of Artificial Intelligence Big Data, Sehan University, Dangjin-si 31746, Republic of Korea; you359@sehan.ac.kr

**Keywords:** wireless time-sensitive networking, time-sensitive networking, time-aware shaper, deep reinforcement learning, wireless LAN

## Abstract

Time-sensitive networking (TSN) technologies have garnered attention for supporting time-sensitive communication services, with recent interest extending to the wireless domain. However, adapting TSN to wireless areas faces challenges due to the competitive channel utilization in IEEE 802.11, necessitating exclusive channels for low-latency services. Additionally, traditional TSN scheduling algorithms may cause significant transmission delays due to dynamic wireless characteristics, which must be addressed. This paper proposes a wireless TSN model of IEEE 802.11 networks for the exclusive channel access and a novel time-sensitive traffic scheduler, named the wireless intelligent scheduler (WISE), based on deep reinforcement learning. We designed a deep reinforcement learning (DRL) framework to learn the repetitive transmission patterns of time-sensitive traffic and address potential latency issues from changing wireless conditions. Within this framework, we identified the most suitable DRL model, presenting the WISE algorithm with the best performance. Experimental results indicate that the proposed mechanisms meet up to 99.9% under the various wireless communication scenarios. In addition, they show that the processing delay is successfully limited within the specific time requirements and the scalability of TSN streams is guaranteed by the proposed mechanisms.

## 1. Introduction

Recently, there has been a rising demand for time-sensitive communication services across both industrial and consumer sectors, with applications ranging from industrial automation to augmented reality and virtual reality [1,2,3]. These time-critical applications are highly sensitive to data transmission delays, which can not only degrade service quality, but also lead to serious accidents [4,5].

In the industrial sector, there has been long-standing research into various real-time network technologies designed for such time-critical applications [6]. Recently, the time-sensitive networking (TSN) standards developed by the IEEE 802.1 working group have garnered attention [7]. Built on Ethernet, which is commonly adopted in local area networks, TSN utilizes the time-aware shaper (TAS) algorithm to control transmission times, meeting the low-latency requirements of time-sensitive (TS) streams [8].

The potential of TSN technologies for time-sensitive communication services has spurred researchers to explore extending this technology into the wireless domain [9,10]. The cost-effectiveness, flexible installation, and ease of maintenance offered by wireless communications are significant advantages [11]. Consequently, research into wireless TSN (WTSN), which leverages wireless communication technologies for time-sensitive networking, is progressing. TSN, a standard at the data link layer, can accommodate various standards from the wireless physical layer, including IEEE 802.11 and 5G [12]. Our research focuses on IEEE 802.11-based WTSN, taking advantage of the cost benefits of unlicensed bands and the ease of integration using the same protocol stack [13]. This paper primarily discusses applying the IEEE 802.1Qbv standard’s TAS to wireless communications.

Applying TSN standard technologies to wireless systems based on the IEEE 802.11 standard involves more than just adopting the TAS algorithm. IEEE 802.11 fundamentally uses a carrier sense multiple access with collision avoidance (CSMA/CA) method for wireless medium access, where devices on the same channel compete for access based on random timing. This unpredictability in CSMA/CA makes it difficult to adhere to the TAS’s transmission gate on/off schedule [14]. Fortunately, the IEEE 802.11 standard also provides mechanisms under the control of the access point (AP) to manage the access times of the stations, allowing the timely transmission of TS streams if the WTSN system model is well-designed to meet TS streams requirements [15,16].

Another challenge in implementing WTSN is the variability of wireless channel conditions depending on the surrounding environment, a core issue addressed in this paper. IEEE 802.11 devices select the optimal bit rate for data transmission based on changing channel conditions, which can make the amount of data that can be transmitted per unit of time inconsistent [17]. Using the fixed traffic scheduling from wired TSN in WTSN could lead to delays in time-sensitive streams. WTSN requires a new scheduling algorithm that considers variations in channel conditions.

Rule-based and optimal solution search algorithms, such as integer linear programming (ILP) and satisfiability modulo theories (SMT), used for generating gate control lists (GCL) in TSN, may not function effectively in the highly variable wireless communication environment [8,18,19]. Rule-based algorithms rely on static rules, making it difficult to adapt to changing conditions, and designing rules that consider all the variable factors in wireless communication is challenging. Optimal solution-based algorithms can consider environmental changes, but as the number of TS streams increases, the time required to find the optimal solution can also increase.

In this paper, we address the issues mentioned by employing reinforcement learning. Reinforcement learning is a method capable of adapting to dynamic environmental changes and deriving optimal scheduling outcomes [20]. We design a wireless TSN model for performing time-sensitive communications in IEEE 802.11 networks and propose WISE: **W**TSN **I**ntelligent **S**ch**E**duler, a reinforcement learning-based WTSN scheduler that can respond to changes in wireless channel conditions.

The main contributions of this paper are as follows:

1. To apply the TAS functionality of TSN to IEEE 802.11-based wireless networks, we design a WTSN network model. In this model, wireless stations can receive exclusive periods from the AP to transmit TS streams. We present potential delay issues caused by changing wireless channel conditions and outline the problem scenarios that the scheduler needs to address.
2. We propose WISE, a deep reinforcement learning (DRL)-based WTSN scheduler that adapts to changes in wireless channel conditions and meets the latency requirements of TS streams. Our DRL framework is designed to learn and adapt to these changing conditions while also learning repetitive stream patterns to satisfy latency requirements in the WTSN model. By comparing variants of WISE, we identify the most suitable DRL model for solving the given problem.
3. Through a comparative evaluation of non-ML schedulers and WISE variants in terms of latency and algorithm processing time, we validate the effectiveness of the proposed WISE and analyze the factors contributing to its performance. Our findings indicate that WISE is the only scheduler that consistently meets the 99.9% latency satisfaction requirement in all scenarios. This achievement, within an acceptable processing time of approximately 95 ms in the evaluated WTSN network environment, is significant compared to the ILP algorithm’s processing time of 3.3 s.

The remainder of this paper is organized as follows: Section 2 discusses related works. Section 3 presents the WTSN network model and the issues arising from changes in wireless channel conditions. In Section 4, we introduce the WISE scheduler for the WTSN network. To demonstrate the advantages of the proposed method, we compare WISE with other schedulers in Section 5. Finally, Section 6 provides the conclusions. The main notations used throughout the paper are summarized in Table 1.

## 2. Related Works

### 2.1. TSN in Wireless Networks

The strengths of TSN technologies in providing time-sensitive communication services have garnered significant attention from many researchers, and numerous studies are still ongoing [4,8]. Recently, this trend has led to attempts to apply TSN technology to the field of wireless communications [11,13,21]. Since the TSN standards is not a single standalone standard, research on its expansion into wireless is also addressing various aspects such as architecture and protocol design [12], time synchronization [9], scheduling [22], and frame replication [23].

In [24], the authors considered the combination of TSN technology with 5G new radio (NR)’s ultra-reliable low-latency communication for providing mission-critical communications, with a focus on industrial and factory automation. They designed architectures and protocols that enable 5G to operate as part of a time-sensitive network, a TSN bridge. Shrestha, D. [25] proposed an enhanced precision time protocol, extending its application to industrial wireless sensor networks from its traditional use for time synchronization in TSN networks. The results of this study confirmed the feasibility of time synchronization over wireless networks. Fang. J. [23], considering the vulnerability of wireless channels to propagation effects, noise, and interference, it was shown that TSN redundancy techniques could be extended from Ethernet to WiFi. This addresses the challenge of maintaining low latency and high reliability in networks faced with unmanaged interference in unlicensed bands.

The TSN standards operates at the link layer, and can use several standard technologies, including wired Ethernet as well as IEEE 802.11 and 5G, as its base media technologies [12]. However, integrating the 5G standard technology with TSN networks may involve complexities, as it requires considering the 5G network as a logical TSN bridge for connection. On the other hand, IEEE 802.11 is based on the same IEEE 802 standard family as IEEE 802.3, facilitating smoother extension of TSN functionalities compared to the 3GPP/5G standards, which are based on a different protocol stack [13]. Aligning with this perspective, we also consider TSN over IEEE 802.11.

### 2.2. Scheduling Time-Sensitive Streams in IEEE 802.11 Networks

As mentioned in the introduction, our main focus is the adaptation of TAS to IEEE 802.11 networks. In the IEEE 802.11 standards, several features that manage the access time of the wireless medium have been discussed [15,16], and there have been studies on transmitting scheduled traffic in IEEE 802.11 networks based on these features.

In [26], a trigger-based multi-user protocol was proposed using the newly mentioned trigger frame in IEEE 802.11ax. This study managed to maintain delays under one millisecond for time-sensitive streams by combining standard technologies such as uplink orthogonal frequency-division multiple access and multi-user enhanced distributed channel access (EDCA) parameter sets. Peón, P. G. [27] proposed a scheduled time medium access control (ST MAC) that configures the gate control mechanism of IEEE 802.1Qbv for use with the IEEE 802.11 chipset, based on the priority queues of IEEE 802.11 EDCA. Implemented on an Atheros AR928X IEEE 802.11 chipset, ST MAC showed improved safety and reduced latency compared to traditional distributed coordination function (DCF)-based MACs. In [28], time-aware scheduling for WiFi 6 was proposed using the newly introduced target wake time along with the trigger frame in IEEE 802.11ax. The static target wake time (TWT)-based time-aware scheduling approach relied on broadcast TWT service periods aligned with the TAS gating cycle to support periodic data and event traffic. The results confirmed that the approach could meet the QoS requirements for both traffic types. Jayabal [22] proposed a transmission gating time hyperchannel (TGT-HC) to support low-latency communications for cyclic control traffic. TGT-HC combines TAS and FIFO scheduling on a contention-free hyperchannel protocol to meet the low-latency requirements of cyclic control traffic. In [29], the scheduling problem of the TSN and WiFi converged network is modeled, and a greedy strategy distributed estimation algorithm is proposed to solve this issue. Compared to the ILP algorithm and the Tabu algorithm, the proposed algorithm is able to maintain time-sensitive communication over WiFi, even as the number of TS flows in the network increases. In [30], the authors propose a DRL-based orthogonal frequency division multiple access (OFDMA) scheduling method that aims to guarantee latency constraints for latency-sensitive stations while maximizing the throughput of high throughput stations.

Most research on adapting TAS functionalities in IEEE 802.11 has concentrated on configuring TAS gate on/off mechanisms. However, some studies often neglect the scheduling of TS frame transmissions in the presence of multiple communication nodes within the shared medium of IEEE 802.11. Additionally, unlike Ethernet-based TSN, which maintains consistent transmission speeds, wireless TSN is sensitive to channel condition changes, resulting in inconsistent transmission speeds. This inconsistency can cause transmission delays, potentially failing to meet the requirements of time-sensitive streams [31]. While solutions are needed for this issue, most research in the wireless domain has focused on maximizing energy efficiency or throughput, with insufficient attention given to transmission delay [32,33,34]. Although the study by Liu et al. [35] considered the age of information to enable real-time communication services in wireless networks, it did not considered characteristics of TS streams in WTSN. Therefore, further research is needed to ensure the proposed algorithm operates effectively in WTSN networks.

Addressing these channel condition changes with traditional rule-based algorithms requires designing specific rules for various scenarios, a task that can be quite complex. Alternatively, as demonstrated in TSN studies [36,37], scheduling problems can be solved using optimal solution search algorithms by incorporating specific scenarios as constraints and conditions. This strategy could be considered for addressing the issues by including dynamic environmental changes in the wireless channel in these algorithms to solve the WTSN traffic scheduling problem. However, this approach could lead to new issues, as the processing time increases with the complexity of the conditions, making it challenging to respond promptly to environmental changes.

## 3. WTSN Network Model and Problems

### 3.1. WTSN Network Model

**Figure 1:**
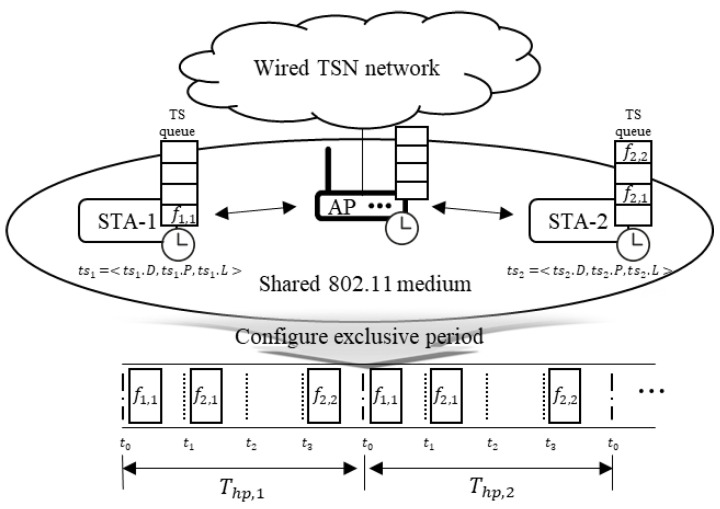
Wireless time-sensitive networking network.

The IEEE 802.11-based WTSN network model is depicted in Figure 1. TS data transmitted over a full-duplex link in the wired network area is sent over a shared medium in the wireless area. Considering this particularity, we assume that nodes using the same channel can register only TS streams of the same transmission direction (uplink or downlink).

In the WTSN network, WTSN nodes (including access points, APs) facilitate time-sensitive communications via TS streams. A TS stream, or flow, entails periodic data transmissions from one sender (talker) to one or more receivers (listeners) [38]. Each TS stream exhibits different data characteristics with specific delay requirements. These data characteristics of the TS stream can be defined as a tuple [39]:

(1) $$ts_{i}=\langle ts_{i}.D,ts_{i}.P,ts_{i}.e2e\rangle ,\;ts_{i}\in TS,$$

where $ts_{i}.D$, $ts_{i}.P$, and $ts_{i}.e2e$ represents the data size, the period of the stream, and the maximum allowed end-to-end latency, respectively.

Considering the connection to wired TSN, a TS stream can traverse the Ethernet link of the wired TSN [40]. Hence, $ts_{i}.e2e$ includes latency requirements from both wireless and wired links. Our primary focus in the WTSN is the delay in the wireless area, denoted as $ts_{i}.L$. Consequently, the WTSN tuple is redefined, with the latency requirement specified solely for wireless links:

(2) $$\begin{array}{cc} ts_{i} & =\langle ts_{i}.D,ts_{i}.P,ts_{i}.L,\rangle \\ \mathrm{where}\;ts_{i}.L & =ts_{i}.e2e-ts_{i}.wired\_latency. \end{array}$$

In TSN, the transmission time of TS frames, which are instances of TS streams, is scheduled on the links of TSN nodes to meet the requirements of each TS stream. The scheduling results are conveyed to the nodes in the TSN network as a gate control list, allowing the nodes to perform time-sensitive communication based on the specified times [8]. Similarly, in WTSN, the transmission time of frames generated from TS streams is scheduled on the links of WTSN nodes that use the same channel. Specifically, for all periodic streams $ts_{i}\in TS$ passing through wireless links, the schedule for each stream needs to be determined.

(3) $$\Psi \left(ts_{i},E\right)=\left\{\left(\phi_{i,j,k}^{start}\right),\left(\phi_{i,j,k}^{end}\right)|f_{i,j,k}\right\},$$

where *E* is set of wireless link, $e_{k}\in E$, and $f_{i,j}$ is the *j*-th frame generated from the *i*-th stream. $\phi_{i,j,k}^{start}$ and $\phi_{i,j,k}^{end}$ are the start and end times of the *j*-th frame transmission in the *i*-th stream from node *k*.

Unlike switched Ethernet, where TSN is commonly applied and provides independent connections between network devices through switch ports, IEEE 802.11 uses the CSMA/CA mechanism for medium access, with nodes sharing the medium. Therefore, scheduling in WTSN must consider these differences.

As mentioned in the introduction, the IEEE 802.11 standard offers features such as point coordination function (PCF), trigger frame, and TWT that provide nodes with exclusive channel access time periods under the management of an AP within the CSMA/CA mechanism. In a shared medium, nodes can be granted exclusive time durations to transmit TS frames. Time synchronization between communication nodes becomes crucial since channel access is timed. Such synchronization can be achieved through beacon signals or fine-timing measurement protocols [41,42].

In the subsequent network model design process, we will examine how to configure the channel for scheduling frames generated from TS streams in a shared medium, based on the features provided by IEEE 802.11.

In the WTSN network, the AP configures an exclusive channel for managing nodes using the period, ts.P, from the tuples of registered TS streams. As defined earlier, a ts stream generates TS data periodically every ts.P. Similar to TSN, WTSN uses the periods of all registered streams to configure and manage an exclusive channel based on a repetitive channel management cycle called the hyperperiod, using the least common multiple of all registered stream periods. The hyperperiod is determined by the following equation:

(4) $$T_{H}=\mathrm{lcm}\left\{ts_{i}.P|ts_{i}\in TS\right\}.$$

In terms of the hyperperiod, $f_{i,j}$ represents the *j*-th frame generated from the *i*-th stream, where $j\in [1,T_{H}/ts_{i}.P]$. Within the hyperperiod, WTSN nodes are allowed to transmit ts frames during a specific time duration, $T_{S}$, within the repeating hyperperiod by the AP.

In our WTSN design, the channel management resolution based on the hyperperiod is $T_{S}$. $T_{S}$ is a parameter set by the WTSN network designer, considering the size of the data to be sent and the data rate of the channel, DR. The network designer also considers the overhead caused by the control data required to send TS data. This overhead can vary depending on the media access technique of the IEEE 802.11 standard and the presence of ACK.

(5) $$T_{S}>\frac{D_{i}}{DR}\;\mathrm{where}\;D_{i}=D_{overhead}+ts_{i}.D.$$

Unlike TSN based on full-duplex Ethernet, nodes in IEEE 802.11-based wireless TSN cannot always occupy the channel due to the competitive nature of channel access. To maximize the efficiency of wireless resource utilization and minimize potential delays, TS data with significantly smaller sizes than $T_{S}$ can be sent sequentially if they share the same destination.

(6) $$T_{S}>\frac{D_{I}}{DR}\;\mathrm{where}\;D_{I}=D_{overhead}+\sum_{i\in I}ts_{i}.D,$$

where *I* represents a set of indices for streams that not only share the same source and destination, but also have identical periods *P*.

Based on the designed model, the WTSN AP manages node access in each $T_{H}$ cycle according to the characteristics of the registered TS streams. The access times of the nodes are managed based on the scheduling results of the TS frames by the WTSN scheduler for each $T_{h,i}$. The AP can support time-sensitive communication by distributing access times to the nodes or triggering exclusive period usage according to the scheduling results. $T_{h}$ is a set of $T_{s}$, and $T_{s,i}\in T_{h}$.

We have modified the scheduling problem to find the schedule for all periodic streams $ts_{i}\in TS$ transmitted over the wireless channel so that each stream is assigned to an appropriate time slot within the $T_{h}$. Therefore, the problem that the scheduler solves in our WTSN network model is as follows:

(7) $$\Psi \left(ts_{i},T_{h}\right)=\left\{\left(\tau_{i,j,k}\right),\left(\phi_{i,j,k}^{start}\right),\left(\phi_{i,j,k}^{end}\right)|f_{i,j,k}\right\},$$

where $\tau_{i,j,k}$ is the time when the TS frame of node *k* enters the queue. Since it is a shared medium, this variable accounts for the delay caused by the frame being queued when node *k* is not granted a time slot.

### 3.2. Problems in WTSN Network

The WTSN scheduling problem can be addressed similarly to the scheduling problem in traditional TSN, by deriving a schedule through optimal solution search-based strategies. We devise the following ILP model to solve the scheduling problem.

*Variables*

- $\phi_{i,j,k}^{\mathrm{start}}$: start time of the *j*-th frame transmission in the *i*-th stream from node *k*.
- $\phi_{i,j,k}^{\mathrm{end}}$: end time of the *j*-th frame transmission in the *i*-th stream from node *k*.
- $\tau_{i,j,k}$: time when the *j*-th frame of the *i*-th stream from node *k* enters the queue.
- $x_{s,k}$: binary variable indicating whether slot $s\in T_{h}$ is allocated to node *k* (1 if allocated, 0 otherwise).
- $\delta_{i,j,k}$: binary variable indicating whether the *j*-th frame of the *i*-th stream from node *k* meets the deadline (1 if met, 0 otherwise).

*Objective Function*

(8) $$\mathrm{maximize}\sum_{i}\sum_{j}\sum_{k}\delta_{i,j,k}.$$

*Constraints*

1. Slot allocation constraint: Each slot $s\in T_{h}$ can be allocated to at most one node *k*.

(9) $$\sum_{k}x_{s,k}\leq 1\;\forall s\in T_{h}.$$

2. Frame non-overlapping constraint: No two frames should overlap in transmission.

(10) $$\phi_{i,j,k}^{\mathrm{start}}\geq \phi_{i^{\prime },j^{\prime },k}^{\mathrm{end}}\;\mathrm{or}\;\phi_{i^{\prime },j^{\prime },k}^{\mathrm{start}}\geq \phi_{i,j,k}^{\mathrm{end}}\;\forall \left(i,j\right)\neq \left(i^{\prime },j^{\prime }\right).$$

3. Deadline constraint: Each frame in the stream must complete transmission within its allowed latency.

(11) $$\phi_{i,j,k}^{\mathrm{end}}-\tau_{i,j,k}\leq ts_{i}.L\;\forall i,j,k.$$

4. Periodic transmission constraint: The frames of each stream must be transmitted periodically according to their period.

(12) $$\tau_{i,j+1,k}=\tau_{i,j,k}+ts_{i}.P\;\forall i,j,k.$$

5. Transmission time constraint: The end time of a frame transmission must be greater than or equal to the start time plus the transmission duration.

(13) $$\phi_{i,j,k}^{\mathrm{end}}\geq \phi_{i,j,k}^{\mathrm{start}}+\frac{ts_{i}.D}{DR}\;\forall i,j,k.$$

6. Slot duration constraint: The total transmission time of all frames in a slot must not exceed the slot duration $T_{S}$.

(14) $$\sum_{i,j}\left(\phi_{i,j,k}^{\mathrm{end}}-\phi_{i,j,k}^{\mathrm{start}}\right)\leq T_{S}\;\forall s\in T_{h},k.$$

As mentioned in the introduction, in WTSN, delays can occur during TS communication due to changes in wireless channel conditions. In IEEE 802.11, communication nodes adapt to changing channel conditions by performing link adaptation (rate control) and adjusting the transmission parameters of the links corresponding to each node. When channel conditions change (especially when they deteriorate), rate control is performed on the links between the AP and nodes to maximize throughput by selecting a lower MCS index, thus reducing the transmission rates [17]. This results in a decreased amount of data that can be sent per unit of time within the $T_{S}$.

In such situations, if scheduling algorithms that do not consider channel conditions are used, the delay requirements of each stream may not be met due to transmission delays within individual $T_{s,i}$ slots, even though the overall network throughput for the registered TS streams is sufficient. In other words, in a wireless environment where channel conditions vary over time, scheduling must consider the varying transmission rates of individual links for each node. To incorporate these considerations into the equation, the scheduling problem is modified as follows:

(15) $$\Psi \left(ts_{i},T_{h}\right)=\left\{\left(\tau_{i,j,k}\right),\left(\phi_{i,j,k}^{start}\right),\left(\phi_{i,j,k}^{end}\right)|f_{i,j,k},DR_{k}\left(\phi_{i,j,k}^{start}\right)\right\},$$

where $DR_{k}\left(\phi_{i,j,k}^{start}\right)$ represents the data rate for each link *k* at the start time of the frame’s transmission, reflecting the dynamic wireless channel conditions.

However, performing adaptive scheduling in WTSN based on channel conditions is not easy. Firstly, changes in wireless conditions are linked to the individual links of each node, making it difficult to design rules that consider the number of nodes and the various MCS indices each node can select. Additionally, simple rule-based algorithms may not be sufficient to meet the registered delay requirements.

An alternative approach is to add the changes in channel conditions as constraints in the previously mentioned optimal solution-based algorithm. However, designing these constraints is also challenging due to the number of nodes and the various MCS indices they can select. Moreover, ILP inherently has a long processing time, making it difficult for the scheduler to quickly produce scheduling results in response to changing channel conditions. The limitations of these scheduling algorithms can be seen in Section 5.

To address these challenges, we aim to explore an approach that uses deep reinforcement learning to learn and adapt to channel changes, producing adaptive scheduling results.

## 4. WTSN Intelligent Scheduler

**Figure 2:**
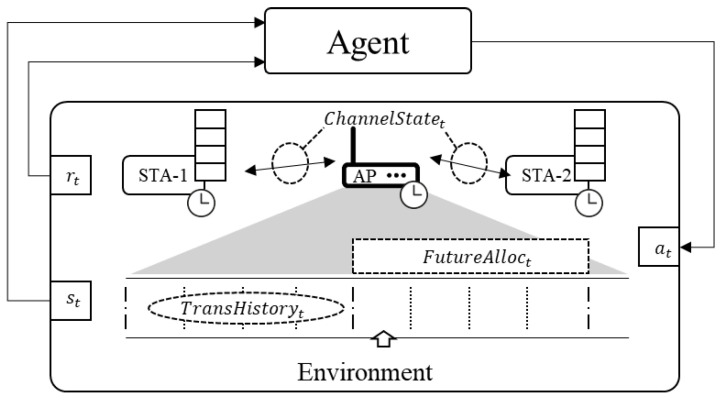
**W**TSN **I**ntelligent **S**ch**E**duler framework.

The proposed WISE framework is illustrated in Figure 2. The WISE agent operates on the WTSN AP. In the RL framework, the agent observes the environment, takes appropriate actions, and learns to maximize rewards. Accordingly, we examine the state, action, and reward designed to enable the WISE agent to derive the scheduling for the given problem.

The state observed from the environment is represented by the following equation, which includes the channel conditions for each node at the current step *t*, the node IDs, the number of frames allocated to slots from the current step *t* to t+i−1, and the transmission history of the last *i* slots before the current step *t*:

(16) $$s_{t}=\left({\mathrm{ChannelState}}_{t},{\mathrm{FutureAlloc}}_{t},{\mathrm{TransHistory}}_{t}\right),$$

where

- ${\mathrm{ChannelState}}_{t}=\left[mcs_{1},mcs_{2},\ldots ,mcs_{N}\right]$, representing the current MCS index for *N* nodes at time *t*, where each $mcs_{k}$ is the MCS index for the node *k*.
- ${\mathrm{FutureAlloc}}_{t}=\left[\left(id_{t},fn_{t}\right),\left(id_{t+1},fn_{t+1}\right),\ldots ,\left(id_{t+i-1},fn_{t+i-1}\right)\right]$, detailing the allocations for the next *i* steps, where each pair $\left(id_{t+j},fn_{t+j}\right)$ consists of the node ID $id_{t+j}$ and the number of frames $fn_{t+j}$ that node is scheduled to transmit in the (t+j)-th step.
- ${\mathrm{TransHistory}}_{t}=\left[h_{t-i},h_{t-i-1},\ldots ,h_{t-1}\right]$, where each $h_{t-k}=\left[fn_{t-k,1},fn_{t-k,2},\ldots ,fn_{t-k,N}\right]$ records the number of frames transmitted by each of the *N* nodes at time t−k, reflecting the transmission history over the past *i* steps.

The channel state information can be obtained from the physical layer, and it is assumed that the AP periodically acquires channel state information through communication with the nodes [43]. The information captures the current MCS index for the link state of each node, allowing the agent to adjust its scheduling decisions based on the condition of $DR_{k}\left(\phi_{i,j,k}^{start}\right)$ in Equation (15), taking into account changes in transmission rates. The ${\mathrm{FutureAlloc}}_{t}$ represents the scheduled transmission plan, indicating the node IDs and the number of frames that each node is scheduled to transmit in each slot for the next *i* steps, including step *t*. This information helps the agent learn the TS stream pattern. The third component of $s_{t}$, ${\mathrm{TransHistory}}_{t}$, is the history of frames transmitted in the past, which the RL agent uses to reference past transmission data and perform actions at step *t*. The ${\mathrm{TransHistory}}_{t}$ has a size of *N* by *i* (the number of past steps to be observed).

The agent determines what action to take at step t based on $s_{t}$. The action $a_{t}$ is defined as:

(17) $$a_{t}\in \left\{1,2,\ldots ,N\right\}.$$

The agent determines which station to allocate at step *t* as its action. In designing the agent, we considered defining the possible actions not only as which node to allocate at step *t*, but also how much TS data to send. Alternatively, actions could be defined to derive a schedule for a set of slots corresponding to a hyperperiod all at once. (This approach might be more intuitive from the perspective of synchronizing with the hyperperiod cycle of the WTSN network). However, these configurations can result in an excessively large action space, leading to issues with the agent’s model convergence [44]. Large action spaces require additional strategies for learning convergence. For the sake of ease of learning, we minimized the action space by selecting only the allocation of stations to a single slot as the action. Configurations based on a large action space remain an open challenge.

The reward received from the environment after performing an action is shown in Equation (18).

(18) $$r\left(t\right)=\sum_{i=1}^{N}\sum_{j\in Q_{i}}-w\left(s_{j}\right)\cdot \mathrm{penalty}\left(\tau_{j},t,L_{j}\right),$$

where:

$$\mathrm{penalty}\left(\tau_{j},t,L_{j}\right)=\left\{\begin{array}{cc} 1 & \mathrm{if}\;\left(t-\tau_{j}\right)\geq L_{j},\\ 0 & \mathrm{otherwise}. \end{array}\right.$$

The components of the equation are defined as follows:

- r(t) represents the reward at step *t*.
- *N* is the number of nodes in the system.
- $Q_{i}$ represents the set of frames in the queue of node *i*.
- $s_{j}$ is the size of frame *j*.
- $\tau_{j}$ is the time when frame *j* entered the queue.
- $L_{j}$ is the maximum allowed latency of frame *j*.
- $w(s_{j})$ is a weight function that adjusts the penalty based on the size of the frame, e.g., $w\left(s_{j}\right)=\frac{s_{j}}{100}$.

To clearly define the goal for the agent, we designed the reward function to count the number of TS frames in all nodes’ queues that exceed the latency requirements as a negative reward at each step. The reward function is designed to directly correlate with the objective function in Equation (8), which represents the scheduling problem’s goal in the ILP model. By minimizing the penalty, the RL agent effectively maximizes the sum of $\delta_{i,j,k}$. The reward function penalizes the agent whenever a frame misses its deadline, thus encouraging the agent to learn policies that maximize the number of frames meeting their deadlines. Additionally, we have introduced a weight function to address the issue where the RL agent more frequently allocates TS streams with smaller TS frame sizes over those with larger TS frame sizes.

Based on the defined state, action, and reward, the RL agent follows the RL framework to learn and perform scheduling. We applied proximal policy optimization (PPO) as the DRL agent. The WISE algorithm based on PPO is outlined in Algorithm 1. The PPO algorithm takes as input the hyperperiod $T_{H}$, the number of nodes *N*, $R_{reset}$ (which resets the environment when the cumulative reward drops below a certain threshold), and $MCS_{list}$ (which defines the range of transmission rates).

The model is trained over a total number of training steps, with the transmission rate for each node’s link being randomly selected from the MCS list at every $T_{H}$. At each step, the agent takes an action, and the total number of frames in the nodes’ queues that fail to meet the stream-defined latency requirements results in a negative reward. This negative reward accumulates in $R_{cum}$, and if it falls below the specific threshold $R_{reset}$, the environment is reset to facilitate model convergence. The remainder of the algorithm’s is identical to the PPO-clip [45].

The computational complexity of the algorithm is calculated by the product of the number of nodes $N_{l}$ in each layer and the number of nodes $N_{l-1}$ in the previous layer of a fully connected neural network model. The total complexity is given by the sum of the computational complexities of all layers,

(19) $$\mathrm{Total}\;\mathrm{Complexity}=\sum_{l=1}^{L}N_{l}\times N_{l-1}.$$

**Algorithm 1** PPO-based Wireless TSN Intelligent Scheduler
 **Require:** Total training steps $T_{\mathrm{total}}$, period $T_{H}$, WTSN nodes *N*, reset reward $R_{\mathrm{reset}}$, $MCS_{\mathrm{list}}$ based on IEEE 802.11 standard. **Ensure:** Optimized policy parameters $\theta^{*}$ and value function parameters $\phi^{*}$.1:Initialize policy parameters θ, value function parameters ϕ.2:Initialize environment and get initial state $s_{0}$.3:Initialize node channel MCS for all nodes *N*.4:
**while** $\;t<T_{\mathrm{total}}\;$ **do**
5:   **if** $t\;\mathrm{mod}\;T_{H}==0$ **then**6:     **for** i=1 to *N* **do**7:        Randomly set channel MCS for node *i* from $MCS_{\mathrm{list}}$.8:     **end for**9:   **end if**10:   Select a node for communication opportunity based on policy $\pi_{\theta }$.11:   Execute action and observe reward $r_{t}$ and new state $s_{t+1}$.12:   $R_{\mathrm{cum}}\leftarrow R_{\mathrm{cum}}+r_{t}$.13:   **if** $R_{\mathrm{cum}}<R_{\mathrm{reset}}$ **then**14:     Reset environment and get new initial state $s_{0}$.15:     $R_{\mathrm{cum}}=0$.16:     Continue to the next iteration of the loop.17:   **end if**18:   Store transition $\left(s_{t},a_{t},r_{t},s_{t+1}\right)$ in rollout buffer D.19:   Compute rewards-to-go ${\hat{R}}_{t}$ and advantage estimates ${\hat{A}}_{t}$ using the value function $V_{\phi }$.20:   **for** epoch=1,…,update_epochs **do**21:     **for** each mini-batch B⊆D **do**22:        Compute ratio $r_{t}\left(\theta \right)=\frac{\pi_{\theta }\left(a_{t}|s_{t}\right)}{\pi_{\theta_{\mathrm{old}}}\left(a_{t}|s_{t}\right)}$.23:        Compute clipped surrogate objective:
$$\begin{array}{cc} L(\theta ) & ={\hat{E}}_{t}[\min(r_{t}\left(\theta \right){\hat{A}}_{t},\;\\ & \;\mathrm{clip}\left(r_{t}\left(\theta \right),1-\epsilon ,1+\epsilon \right){\hat{A}}_{t})]. \end{array}$$24:        Update θ by maximizing L(θ) via stochastic gradient ascent.25:        Update ϕ by minimizing the MSE loss between ${\hat{R}}_{t}$ and $V_{\phi }\left(s_{t}\right)$.26:     **end for**27:   **end for**28:   t←t+1.29:
**end while**



Given the input dimension $d_{in}$, the hidden layer dimension *H*, and the output dimension $d_{out}$, the total computational complexity is expressed as follows:

(20) $$O\left(H\times d_{in}+\sum_{i=2}^{L-1}H_{i}\times H_{i-1}+d_{out}\times H_{L-1}\right).$$

In our WTSN network, the agent aims to reschedule every $T_{H}$ cycle as the rate changes. According to the computational complexity, the processing time for deriving the schedule is related to the sizes of the input and output dimensions, i.e., $|s_{t}|$ and $|a_{t}|$. This implies that the network architecture, including the number of layers and the dimensions of each layer, should be carefully selected to ensure efficient scheduling, taking into account the state and action space parameters.

## 5. Evaluation

To evaluate the proposed WISE, we conducted a comparison between non-ML algorithms and WISE. For non-ML algorithms, we selected earliest deadline first (EDF), credit-based scheduler (CBS), and ILP algorithms. The EDF algorithm selects the node with the shortest deadline for the first frame in each communication node’s queue at every step. We also included a modified version of EDF called weighted EDF (WEDF), which assigns higher weights to nodes with larger amounts of queued frames. In WEDF, the weight is simply calculated by multiplying the deadline by the reciprocal of the total length of frames in the queue, making nodes with more frames more likely to be selected.

The CBS is adapted from the credit-based shaper [46] commonly used in TSN and applied to our WTSN network. In this scheduler, at each step, the node with frames to send and the highest credit (with credit > 0) is selected. The credit increases as the frame waits to be sent and decreases when the frame is sent, with the rate of increase and decrease set equal to the data rate of that step. The ILP algorithm was previously mentioned in Section 3. Although EDF and CBS algorithms cannot instantly know the deadlines of frames or the credit of communication nodes in real situations, we assumed they could for the purpose of deriving scheduling results.

Performance evaluation first compared the latency requirement satisfaction rates of non-ML models and WISE models (Section 5.2). We constructed WISE algorithms based on PPO, as well as deep Q-Learning (DQN) and advantage actor-critic (A2C), to analyze which DRL model adopted by WISE performs best under varying channel conditions. This analysis focused on how the characteristics of each DRL model influence the achievement of the given objectives.

Based on the DRL model with the highest performance, Section 5.3 compares the empirical cumulative distribution function (ECDF) of latency for both ST-A and ST-B under varying channel conditions between non-ML models and WISE. The ECDF helps infer the probability that a random variable will not exceed a certain value through repeated trials. This allows us to verify if at least 99.9% of the frames generated in the scenario meet the delay requirements and observe the impact of scenario changes on the scheduling results for different stream types through a comparison of their ECDF graphs. At this point, we compared two versions of WISE, WISE with MCS and WISE without MCS (WISE w/o MCS), to determine the importance of considering channel conditions in the MDP design.

Finally, the evaluation of algorithm processing time addresses whether the algorithms can immediately derive scheduling results in response to changing channel conditions in Section 5.4. Under the assumption that the WTSN network manages the network in hyperperiod cycles, the ability to produce scheduling results within this time period is crucial for the evaluation.

### 5.1. Evaluation Scenarios and Simulation Setup

The specific configuration of the IEEE 802.11-based WTSN network used in this experiment is detailed in Table 2. The network comprises four WTSN nodes registering TS streams to a single AP. In our WTSN network environment, the medium access method used by WTSN communication nodes is the PCF [47]. Under the control of the AP, based on beacon signals, WTSN nodes receive contention-free, exclusive access time slots, enabling the transmission of time-sensitive frames. Based on the hyperperiod configuration discussed in Section 3, the AP allocates scheduled time slots using PCF, with each time slot duration $T_{S}$ set at 1 ms and the hyperperiod $T_{H}$ determined by the stream periods 10, 100 set at 100 ms. In our WTSN network configuration using PCF, the transmission overhead consists of SIFS and a poll frame, which are, respectively, 16 microseconds and 22 bytes.

**Figure 3:**
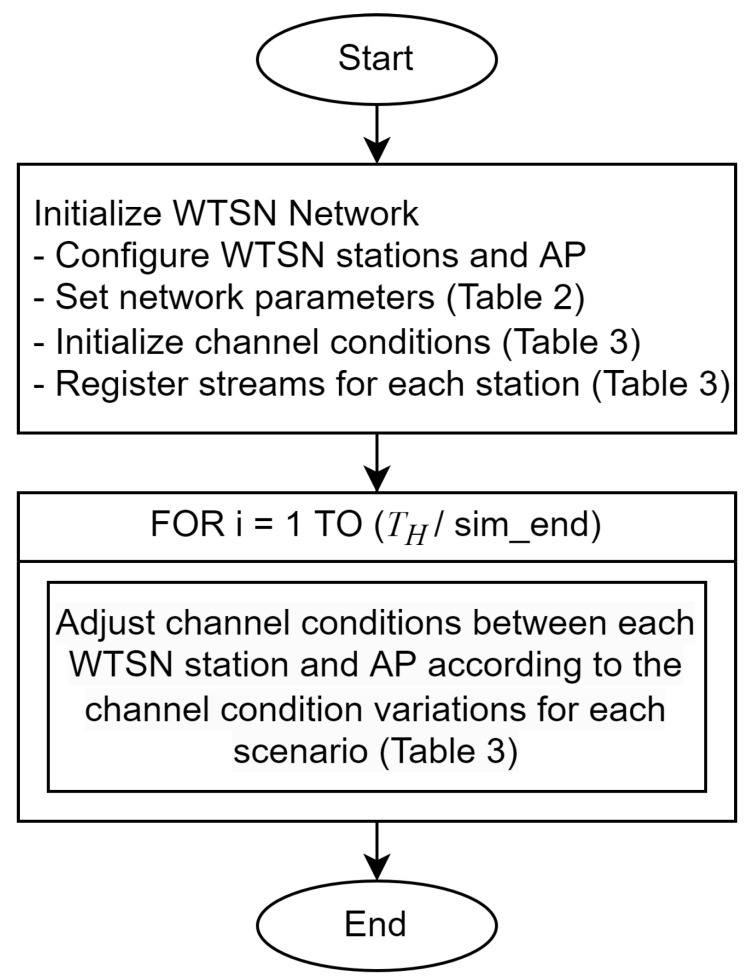
Scenario flowchart.

For the experiments, we designed evaluation scenarios for three different channel conditions and number of streams. The detailed configurations of the scenarios are provided in Table 3, and the flowcharts of the scenarios are illustrated in Figure 3. Two nodes register a type A stream (ST-A), and the other two nodes register a type B stream (ST-B).

In each scenario, after the WTSN network is initialized, the channel conditions between each WTSN station and the AP change every $T_{H}$ until the simulation ends. Streams are registered, and frames begin to be generated at each node according to the scheduling results derived from the ILP algorithm using the initial channel condition values. Each stream type has different delay requirements of 3 ms and 10 ms, respectively, and frames are generated repetitively at defined periods from the moment the first frame is transmitted. The timing of stream registration and frame generation varies with each simulation. We performed 10 simulations for each of the three scenarios. Frame delay data for the first $T_{H}$ cycle of this initial period are excluded.

The first scenario simulates a constant channel condition over 10 s with a fixed MCS index of 6, serving as an ideal baseline to observe the ECDF delay distribution for each algorithm without channel variation. The second scenario involves sequential changes in channel conditions for each node over the same duration, with the MCS index decreasing from 4 to 2 and then increasing back to 4, simulating environments like factories with moving objects. In the third scenario, all nodes experience a simultaneous gradual reduction in channel conditions, with the MCS index dropping from 3 to 1 over 10 s, representing a deteriorating network environment due to factors like dust.

In developing and implementing WISE, we adopted DQN, A2C, and PPO models using Gymnasium [48] and Stable-Baselines3 [49]. The corresponding model parameters are detailed in Table 4. Since the agent uses state information comprising channel status for four WTSN nodes, future allocation for the last 10 time slots, and transmission history from the past 10 slots, the input layer consists of 4+10×2+4×10=64 units. WISE is also evaluated in a version that does not consider MCS in the state, referred to as WISE w/o MCS, which has a state size of 60. The output layer assigns one of four stations, thus it includes four units. We conducted training over 1,000,000 total steps. During training, if the cumulative reward (negative reward) of a single episode drops below 0.1% of the total number of frames generated in the scenario (total frames × 0.001), the environment is reinitialized. For WISE-DQN, which is prone to significant Q value estimation errors, we used Huber Loss, while WISE-A2C and WISE-PPO employed mean squared error (MSE) as the loss function. The replay buffer size for WISE-DQN is 1,000,000, and the target update interval is set to 100, corresponding to the $T_{H}$. The clip range for PPO is 0.2. The remaining parameters for each model followed the default settings in Stable Baseline3. To accommodate learning about changes in channel conditions, the MCS level of each node in the environment is randomly set between 0 and 9 at each hyperperiod.

### 5.2. Evaluation of Latency Requirement Satisfaction Rate

In this evaluation, we compared the satisfaction rate of the latency requirements for all frames generated from ST-A. Overall, the performance ranking was as follows: WISE-PPO, WISE-A2C, WEDF, EDF, ILP, CBS, and WISE-DQN (see Table 5 for details). The WISE-PPO was the only algorithm to meet the requirements for over 99.9% of the frames in all scenarios.

Analyzing the results for non-ML algorithms, WEDF performed the best, while EDF and ILP showed similar performance, and CBS performed the worst. EDF struggled significantly in scenario 3. As resources became scarce over time, the scheduling approach that only considered deadlines performed poorly. WEDF performed better in scenario 3 because nodes with more queued frames had higher selection priority based on their weight. ILP’s performance declined as the channel conditions worsened in scenario 3, as it could not account for changing conditions. CBS performed reasonably well up to scenario 2, but faltered in scenario 3. In situations where many nodes were waiting due to limited channel resources, multiple nodes often had the same positive credit, leading to increased frame delays as more nodes were left unselected in each slot.

Comparing DRL-based WISE, WISE-DQN showed poor performance, achieving only 93.4% even in scenario 1, indicating it failed to learn the given objectives. Scenario 1 involves constant channel conditions, so even with the same scheduling results each cycle, a 100% latency satisfaction rate could be achieved. Poor performance in this scenario indicates that WISE-DQN failed to learn the repetitive periodic frame transmission patterns of the TS streams registered in the WTSN network. It is inferred that it failed to learn these patterns because the random sampling of stored transitions in the replay buffer likely prevented effective learning.

WISE-PPO and WISE-A2C, as shown in the results table, effectively learn the traffic patterns of TS streams, unlike WISE-DQN. PPO and A2C, which collect data on relatively short transitions in a rollout buffer for batch learning, demonstrate advantageous results in learning repetitive patterns. Among the compared algorithms, WISE-PPO and WISE-A2C exhibited the highest performance; however, only WISE-PPO met the 99.9% threshold in all scenarios, whereas WISE-A2C fell short in scenarios 2 and 3. This comparison highlighted that the stability provided by PPO’s clipping mechanism led to better scheduling results.

### 5.3. Comparative Evaluation of Latency ECDF

In this section, we compare the ECDF latency of both ST-A and ST-B to examine how channel variations and the schedules generated by different algorithms affect the transmission delays of each stream’s frames. The comparison includes the PPO-based WISE (WISE with and w/o MCS), EDF, CBS, and ILP algorithms. To focus on the comparison of basic algorithms, WEDF is excluded from this comparison. The latency resolution of the ECDF results graph is set at 0.1 units, with type A ranging from 0 to 3 and type B from 0 to 10.

#### 5.3.1. Evaluation Results in Scenario 1

**Figure 4:**
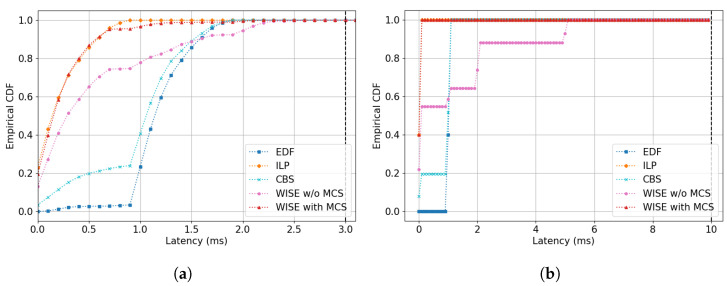
ECDF graph of latency in Scenario 1: (**a**) Stream Type A. (**b**) Stream Type B.

In Scenario 1, due to no change in channel conditions, it is observed that all algorithms achieve delay within the 99.9% probability bound (delays up to 3 are 100%); please see Figure 4a,b. EDF shows fewer occurrences of delay in the 0 to 1 range after the initial few frames, which can be attributed to the competition between nodes registered with low-latency requirement streams (nodes 1 and 2). This competition is also evident in the ST-B graph, where despite not reducing the per $T_{S}$ resources, nearly all frames in EDF experience a delay at the 1 mark.

The competition is similarly observed in CBS, though to a lesser extent compared to EDF. In ST-A, the proportion of delays under 1 s is higher than 0.2, and a similar pattern is seen in the ST-B results, where the proportion of delays under 1 s is 0.2. Since CBS assigns credits to waiting nodes based on the value of their credit, it ultimately allows nodes with ST-A and nodes with ST-B to have an opportunity to send frames in the order they have waited.

ILP results show that all frames for ST-A fall within the 0 to 1 delay range. The shape of the graph represents a cumulative distribution function of an exponential distribution, decreasing as the delays increase. This is an ideal graph shape, since channel conditions do not change. The results for ST-B stream, shown in Figure 4b, are similar (due to fewer streams, all frames show a 100% delay probability distribution at 0.2 or less).

The WISE algorithm, particularly WISE with MCS, which includes channel condition information in its state, shows a graph shape almost similar to ILP. However, WISE without MCS sees about 25% of frames experiencing delays at later time points. Both models, being ML-based, find it challenging to display ideal probability distribution graphs like ILP because they do not repeat the same scheduling result at each time step cycle, as long as the stream’s latency requirements are met without incurring a negative reward. Nevertheless, WISE with MCS, which can account for individual node channel condition changes every $T_{H}$, shows a result graph similar to ILP. This similarity can be interpreted as recognizing from the state information that there is no change in channel conditions. This is also evident in ST-B’s results, closely following ILP’s graph, indicating that the agent effectively utilizes channel condition change information.

#### 5.3.2. Evaluation Results in Scenario 2

**Figure 5:**
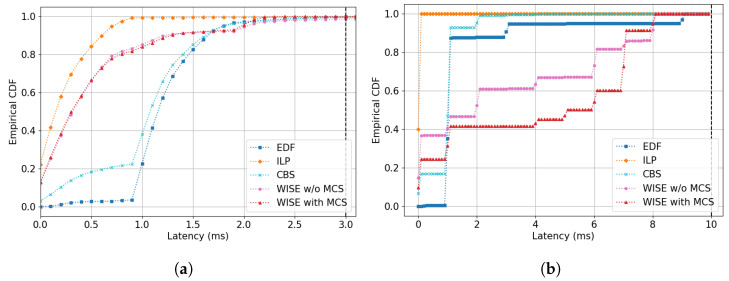
ECDF graph of latency in Scenario 2: (**a**) Stream Type A. (**b**) Stream Type B.

The ECDF graph for ST-A in Scenario 2, shown in Figure 5a reveals that, apart from the WISE with MCS algorithm, the variations in other algorithms were not significantly different compared to Scenario 1. This scenario involves changes in channel conditions; however, only one node experiences changes in channel conditions during the period $T_{H}$, coupled with a temporary decrease and subsequent return in MCS index. Hence, it is assumed that there was no substantial difference in overall network resources compared to Scenario 1, and the changes in the graph did not dynamically differ from those in Scenario 1. However, a closer look at the distribution after the 90% mark shows minor but noticeable differences in other algorithms as well. In Scenario 2, EDF shows more than 3% occurrences of delays exceeding 2, a distribution absent in Scenario 1, and for a delay requirement of 3, it achieves 99.5%, slightly below the 99.9% threshold. Similarly, CBS also has delays exceeding 2 s in more than 3% of cases, with 99.5% of delays under 3 s, which is below the 99.9% threshold. The ST-B graph indicates about 16% of frames are transmitted with a delay of 1 or more, suggesting changes due to channel conditions. In Figure 5b, CBS shows that approximately 93% of frames have delays under 1.2 s, compared to the previous 100%, reflecting the impact of resource shortages due to channel changes. ILP, which schedules based on initial channel conditions, maintains a graph shape similar to Scenario 1. Yet, unlike Scenario 1, where no delays over 1 occurred, about 0.1% of frames show delays exceeding 1 in Scenario 2, and for a delay requirement of 3, it reaches 99.4%, failing to meet the 99.9% standard. WISE with MCS and WISE w/o MCS satisfy the delay requirement of 3 at 99.9% and 98.4%, respectively, with only WISE with MCS achieving 99.9% satisfaction in Scenario 2. The graph indicates a shift towards a more skewed distribution for WISE with MCS compared to Scenario 1. Nodes were sequenced based on changes in channel conditions to prevent delays exceeding 3, resulting in an increased frame distribution in the 1–3 delay range. This scheduling change allowed WISE with MCS to meet delay requirements even when other algorithms failed to reach 99.9%. This is evident in the ST-B graph where, unlike in Scenario 1, 70% of frames are transmitted with a delay of at least 0.2. WISE w/o MCS, despite dynamically adjusting its scheduling more than in Scenario 1, performed the worst due to lack of information on channel conditions.

#### 5.3.3. Evaluation Results in Scenario 3

**Figure 6:**
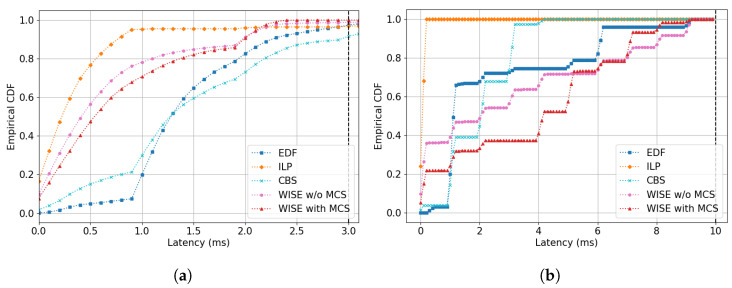
ECDF graph of latency in Scenario 3: (**a**) Stream Type A. (**b**) Stream Type B.

Scenario 3 depicts a situation where the channel conditions for all nodes gradually deteriorate, with the MCS level decreasing from 3 to 1, resulting in resource allocation per time slot being halved towards the latter part of the simulation period. As a result, all algorithms, except WISE with MCS, which maintained 99.9% performance, exhibited more than 1% of frames experiencing delays greater than 3 as shown in Figure 6a. Examining EDF specifically, there is a noticeable increase in the distribution of delays between 2 and 3 compared to Scenarios 1 and 2. CBS showed similar results. The distribution of delays within 3 stands at 96.4%, indicating that the reduction in the amount of data that can be transmitted per slot leads to frames continuously accumulating in the queue, thereby resulting in cumulative transmission delays. CBS performed worse, with 92.7% of delays within 3 s. The increase in frame accumulation due to node competition is also evident in the 0–1 interval of the graph, which saw a significant increase in distribution compared to previous scenarios where this range was nearly absent. This suggests that increased competition among nodes caused frames in the queue to be transmitted in large quantities when given the opportunity. In CBS, competition with ST-B intensified due to the same positive credit values, resulting in performance drop in this scenario, as indicated by the increased distribution in the 2–4 s range for ST-B as shown in Figure 6b.

Similarly, the ST-B graph shows an increase in later delay times compared to Scenarios 1 and 2, highlighting the same underlying causes. ILP, with frame delays extending up to 5.3 ms, only achieved a distribution up to 96.4% for delays within 3, suggesting that the algorithm struggles to adapt dynamically to changing channel conditions based on its initial scheduling decisions.

WISE with MCS and WISE w/o MCS recorded delay frequencies of up to 3 at 99.9% and 98.6%, respectively. Observing the graph’s curve changes, both algorithms show longer transmission delays for frames compared to Scenarios 1 and 2. Notably, WISE w/o MCS in Scenario 3 showed an improvement to 98.6% from 98.4% in Scenario 2 for delays within 3, suggesting it can somewhat infer channel condition changes based on the number of frames generated and the transmission history, even without specific channel condition information.

However, to achieve a high performance level of 99.9%, it is evident that explicit information about channel conditions, as provided in WISE with MCS, is necessary. From the results in the ST-B graph, it appears that WISE with MCS dynamically adjusted the frequency of delays, possibly by reallocating transmission opportunities from nodes under ST-B, which had more lenient delay requirements, to those under ST-A to meet the stricter delay requirements.

### 5.4. Evaluation of Processing Time

In this section, the processing time of the WISE algorithm was evaluated by comparing it with other algorithms as the number of streams increased. In this experiment, the stream sets were randomly generated with 100, 200, …, up to 500 streams. The configuration of the WTSN network and the stream types present within the network were identical to previous experiments, with two streams each of ST1 and ST2.

**Figure 7:**
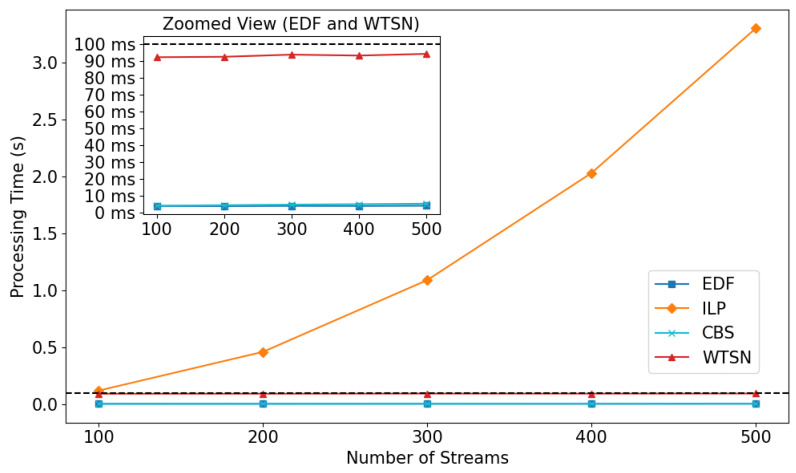
Processing time graph according to number of streams.

WISE, EDF, and CBS calculated the total time taken to derive scheduling results for one hyperperiod cycle, i.e., the sum of the time taken to proceed through 100 time slots. The ILP algorithm measured the time taken to find the scheduling solution. The results are shown in Figure 7.

The graph illustrates two views: one with a time unit of seconds to show all three algorithms, reflecting the high processing time of ILP, and a zoomed-in view with a time unit of milliseconds for EDF, CBS, and WISE. According to the experimental results, unlike ILP, whose processing time increases exponentially with the number of streams, WISE, EDF, and CBS do not show a significant increase in processing time as the number of streams increases. WISE, EDF, and CBS all complete processing within 100 ms even as the number of streams increases (approximately 95 ms, 4 ms, and 5 ms, respectively, for 500 streams). Unlike EDF, the processing time for CBS slightly increases as the number of streams grows. This is attributed to the additional time required to calculate credits as the number of frames to be sent by the selected nodes increases. All three algorithms can derive scheduling results within one hyperperiod cycle suggests that they can adapt to changing channel conditions and support time-sensitive communication in sync with the exclusive channel management cycle. However, ILP takes approximately 3.3 s (equivalent to 33 cycles) for 500 streams, making immediate response to channel changes impractical.

## 6. Conclusions

In this paper, we designed an IEEE 802.11-based WTSN network model to support time-sensitive communication services in the wireless domain. In this model, communication nodes in the WTSN model are granted exclusive channel access to transmit registered TS streams under the control of the AP. We highlighted the delay issues caused by reduced resources per slot due to changes in the wireless channel environment and proposed WISE, an intelligent scheduler based on deep reinforcement learning, to maintain time-sensitive communication services despite these changes.

We first compared three variants of WISE (WISE-PPO, WISE-A2C, WISE-DQN) and three non-ML models (EDF, rule-based; CBS, rule-based; ILP, optimal solution-based) in terms of latency requirement satisfaction rates. According to our experiments, WISE-PPO was the only algorithm that met the latency requirements across all three scenarios. The non-ML models exhibited significant performance degradation as channel conditions worsened, with EDF, CBS, and ILP achieving 96.4%, 92.7%, and 96.4% performance, respectively, in the most challenging scenario. Despite WISE-DQN being provided with state information capable of learning the environment, it did not perform well because it failed to learn the repetitive stream patterns of the WTSN. Although WISE-A2C showed high performance, it could not achieve the 99.9% threshold in inference due to a lack of stability in training compared to WISE-PPO.

To conduct a more detailed comparative evaluation of the algorithms, we examined and compared the ECDF graphs of the delays for two types of streams across different scenarios in the same environment. We analyzed both WISE with MCS, which directly receives channel state information, and WISE without MCS. The results indicated that WISE’s superior performance resulted not only from receiving direct channel state information as part of its state, but also from implicitly learning the relationship between the delay requirements of ST-A and ST-B.

In the final experiment, we evaluated the processing time of the comparison algorithms as the number of streams increased to determine if they could produce immediate scheduling results in response to channel changes. The results showed that WISE maintained an acceptable processing time of approximately 95 ms even with an increase to 500 streams. In contrast, ILP’s processing time increased exponentially with the number of streams, taking about 3.3 s.

In conclusion, the proposed WISE demonstrates its potential as an adaptive scheduler for wireless TSN by meeting delay requirements and maintaining acceptable processing times across various wireless communication scenarios. We anticipate that our WTSN model and WISE algorithm will contribute to providing wireless time-sensitive communication services for time-critical applications in both industrial and consumer sectors.

## Figures and Tables

**Table 1:** Description of notations.

Notation	Definition
TS	Set of time-sensitive streams (flows)
$ts_{i}$	*i*-th stream in set TS; each ts is defined <ts.D,ts.P,ts.L>
$f_{i,j}$	frame generated in the *i*-th stream; $j\in [1,T_{H}/ts_{i}.P]$
$T_{H}$	Time duration of hyperperiod
$T_{S}$	Time duration of a time slot within the hyperperiod
$T_{h,i}$	The *i*-th hyperperiod of hyperperiod cycles
$T_{s,i}$	The *i*-th time slot in $T_{h}$
$\phi_{i,j,k}^{start}$	The start time of the *j*-th frame transmission in the *i*-th stream from node *k*
$\phi_{i,j,k}^{end}$	The end time of the *j*-th frame transmission in the *i*-th stream from node *k*
$\tau_{i,j,k}$	Time when the *j*-th frame of *i*-th stream from node *k* enters the queue
s(t)	The state at step *t*
a(t)	The action at step *t*
r(t)	The reward at step *t*

**Table 2:** IEEE 802.11 based WTSN network parameters.

Parameter	Value
Medium Access Method	Point Coordination Function
Channel Bandwidth	20 MHz
Spatial Stream	1
Guard Interval	800 ns
Slot Size $T_{S}$	1 ms
Hypercycle $T_{H}$	100 ms
Tx Overhead	SIFS: 16 μs
	Poll Frame: 22 byte

**Table 3:** Configurations for WTSN communication scenarios.

Scenario	Channel Condition Variation	Num of Streams	Stream Type A (ST-A)	Stream Type B (ST-B)
S1	Constant channel conditions MCS6	ST-A: 400/400 ST-B: 50/50	Data Size: 100 byte Period: 10 ms Latency: ≤ 3 ms	Data Size: 1000 byte Period: 100 ms Latency: ≤ 10 ms
S2	Sequential changes in channel conditions MCS4->MCS2->MCS4 per each STA	ST-A: 300/300 ST-B: 40/40		
S3	Overall decline in channel conditions MCS3->MCS1	ST-A: 200/200 ST-B: 30/30		

**Table 4:** Model parameters.

Parameters	WISE-DQN	WISE-A2C	WISE-PPO
Dimension of input layer	4+(10×2)+(4×10)=64		
Dimension of output layer	4		
Number of neurons in two hidden layers	64, 64		
Total Time Step	1,000,000		
Reset reward	Total frames × 0.001		
Learning rate	0.001		
Batch size	64		
Discount factor	0.99		
optimizer	Adam		
Loss function	Huber Loss	MSE	MSE
Replay Buffer	1,000,000	-	-
target update interval	100	-	-
Number of epoch	-	-	10
Clip range	-	-	0.2

**Table 5:** Latency requirement satisfaction rate of stream type A.

	Non-ML				WISE		
	EDF	WEDF	ILP	CBS	DQN	A2C	PPO
Scenario 1	100%	100%	100%	100%	93.4%	99.9%	100%
Scenario 2	99.5%	99.6%	99.4%	99.5%	91.3%	99.6%	99.9%
Scenario 3	96.4%	98.8%	96.4%	92.7%	90.5%	99.4%	99.9%

## Data Availability

Data are contained within the article.
